# Combination of In Situ Feeding Rate Experiments and Chemical Body Burden Analysis to Assess the Influence of Micropollutants in Wastewater on *Gammarus pulex*

**DOI:** 10.3390/ijerph16050883

**Published:** 2019-03-11

**Authors:** Sarah Könemann, Yvonne Müller, Daniel Tschentscher, Martin Krauss, Pedro A. Inostroza, Ira Brückner, Johannes Pinnekamp, Sabrina Schiwy, Henner Hollert

**Affiliations:** 1 Department of Ecosystem Analysis, Institute for Environmental Research, RWTH Aachen University, Worringerweg 1, 52074 Aachen, Germany; yvonne.mueller@bio5.rwth-aachen.de (Y.M.); daniel.tschentscher@rwth-aachen.de (D.T.); s.schiwy@bio5.rwth-aachen.de (S.S.); 2 Department Environmental Toxicology, Swiss Federal Institute of Aquatic Science and Technology, Eawag, Überlandstrasse 133, 8600 Dübendorf, Switzerland; 3 Department Effect-Directed Analysis, Helmholtz Institute for Environmental Research-UFZ, Permoserstrasse 15, 04318 Leipzig, Germany; martin.krauss@ufz.de (M.K.); pedro.inostroza@bioenv.gu.se (P.A.I.); 4 Department of Biological and Environmental Sciences, University of Gothenburg, PO BOX 461, 40530 Gothenburg, Sweden; 5 Waterboard Eifel-Rur, Eisenbahnstrasse 5, 52353 Düren, Germany; Ira.Brueckner@wver.de; 6 Institute of Environmental Engineering, RWTH Aachen University, Mies-van-der-Rohe-Strasse 1, 52074 Aachen, Germany; pinnekamp@isa.rwth-aachen.de

**Keywords:** body burden analysis, feeding rate inhibition, *Gammarus pulex*, in situ monitoring, internal concentrations, micropollutants, wastewater, wastewater treatment

## Abstract

Wastewater discharge is one of the main sources of micropollutants within the aquatic environment. To reduce the risks for the aquatic environment, the reduction of the chemical load of wastewater treatment plant effluent is critical. Based on this need, additional treatment methods, such as ozonation, are currently being tested in several wastewater treatment plants (WWTPs). In the present study, effects were investigated using in situ feeding experiments with *Gammarus pulex* and body burden analyses of frequently detected micropollutants which used a Quick Easy Cheap Effective Rugged and Safe (QuEChERS) multi-residue method to quantify internal concentrations in collected gammarids. Information obtained from these experiments complemented data from the chemical analysis of water samples and bioassays, which predominantly cover hydrophilic substances. When comparing up- and downstream feeding rates of *Gammarus pulex* for seven days, relative to the WWTPs, no significant acute effects were detected, although a slight trend of increased feeding rate downstream of the WWTP Aachen-Soers was observed. The chemical load released by the WWTP or at other points, or by diffuse sources, might be too low to lead to clear acute effects on *G. pulex*. However, some compounds found in wastewater are able to alter the microbial community on its leaves, leading to an increase in the feeding rate of *G. pulex*. Chemical analysis of internal concentrations of pollutants in the tissues of collected gammarids suggests a potential risk for chronic effects with the chemicals imidacloprid, thiacloprid, carbendazim, and 1H-benzotriazole when exceeding the critical toxic unit value of −3. This study has demonstrated that a combination of acute testing and measurement of the internal concentration of micropollutants that might lead to chronic effects is an efficient tool for investigating river systems, assuming all relevant factors (e.g., species or season) are taken into account.

## 1. Introduction

In recent years, the occurrence of emerging contaminants in the aquatic environment has increasingly been reported by the scientific community and has become an environmental issue of global concern [1,2,3]. Emerging contaminants, also referred to as micropollutants, are mainly of anthropogenic origin and consist of various different groups of chemicals, such as pharmaceuticals, personal care products (PCPs), fragrances, steroid hormones, industrial chemicals, and pesticides [1,4]. Micropollutants commonly occur at trace concentrations in the lower μg/L or ng/L range [5]. These low concentrations in combination with suspected low toxic effect concentrations and the large variety of micropollutants that are likely to be released into the environment not only leads to increased efforts needed for detection and chemical analysis but also challenges current drinking water and wastewater treatment processes [6]. In industrialized countries, such as Germany, more than 90% of incoming wastewater is treated in centralized wastewater treatment plants (WWTPs) [5]. However, as current conventional WWTPs are designed to eliminate nutrients in the mg/L range, micropollutants are not sufficiently removed by secondary and tertiary treatment methods [7]. Consequently, WWTP effluents are a major point source for trace contaminants in surface water [8,9], which leads to complex mixtures of micropollutants being discharged into surface and ground waters [9]. Since the exact composition of WWTP effluents is predominantly unknown and can vary over time, and chemical analysis requires a substantial amount of a priori knowledge about the substances that could be present, in vivo, in vitro, and in situ effect-based methods (EBMs) should be used to complement chemical analysis in ecotoxicological risk assessment [10,11].

Laboratory toxicity assays have been used for many years to determine the ecotoxicological effects of chemicals [12,13]. However, explanatory power of these toxicity studies is limited due to differences in exposure conditions between the laboratory and the field [14,15]. Thus, uncertainties are associated with extrapolation of results to the natural environment. As a link between the laboratory and the field, in situ bioassays can help to overcome these uncertainties by lowering extrapolation errors [16,17]. In addition to providing more realistic exposure environments, field exposures reduce the risk of artefacts occurring through sample collection and processing [18]. Since the early twentieth century, biomonitoring has been used to assess the water quality of rivers and streams [19]. The most commonly used biomonitoring approaches are based on benthic macroinvertebrates, which are also the base for several biotic indices [20,21]. With most of these methods, changes in community structure are investigated, which reflect integrative effects from diverse sources. However, this diversity of sources and environments makes it complicated to identify direct relationships between cause and effect [22]. To be able to link chemical contamination to observed environmental effects, complementary approaches that are based on investigation of lower levels of organisation, like single species tests, should be used [22]. However, since single species tests only show the sensitivity of the chosen species, it would be beneficial to extend the test system by including multi-species or even community-based tests in risk assessment and regulation if an effect was detected at the single species level [23]. As one of the responses to chemical contamination that can be determined, the inhibition of feeding is of particular interest for multi-scale water quality assessment [22]. Feeding is an endpoint of ecological concern as it is associated with fitness and life history traits of organisms [24,25,26]. In the case of aquatic invertebrates, feeding rate inhibition is often one of the first observed responses to chemical contamination and can be triggered by various groups of chemical compounds, such as metals, insecticides, and pharmaceuticals [27,28]; gammarids in particular are prone to exposure to these kinds of chemicals .

Gammarids are crustaceans, within which they form a sub-order of the amphipods [29]. Among the amphipods, the genus *Gammarus* has the greatest number of species, and those species are native to mainly freshwaters of the northern hemisphere. *Gammarus* are often found under rocks, in sandy or coarse substrates, and under leaves, where they predominantly occur in high abundance and, thus, often dominate macroinvertebrate communities [30]. As benthic organisms, gammarids spend significant periods of their life cycle in close contact with the sediment and the water column above [31]. Consequently, gammarids are steadily exposed to not only water-soluble substances in the water column but also to hydrophobic substances that are adsorbed to the sediment and their diet [32]. These hydrophobic compounds are taken up by the gammarids predominantly via food and accumulate in their tissues [33]. Due to the long lifespans of gammarids in combination with low environmental concentrations of substances that elicit sublethal effects, the accumulation of such compounds is possible over a long time [34]. Hence, biota act as a kind of passive sampler for bioavailable compounds [35] and can be used as time-integrative tool in contamination assessment. The extraction of gammarid tissues and lipids with subsequent analysis provides an indication of the internal concentration of compounds, which is a more suitable surrogate for biologically effective concentration than external concentrations [36]. A promising analytical tool is the Quick Easy Cheap Effective Rugged and Safe (QuEChERS) multi-residue method, which was originally developed to extract and recover residues of pesticides from food matrices, such as fruits or vegetables [37,38]. With QuEChERS, it is possible to effectively examine a wide range of compounds, including highly polar as well as highly acidic and basic ones [38].

The aim of the present study was to bioanalytically evaluate the ecotoxicological state of the River Wurm and to determine the ecotoxicological impact of treated wastewater released predominantly by the WWTP Aachen-Soers and the smaller WWTP Eilendorf into the receiving streams. Therefore, in this study, an in situ feeding inhibition experiment was combined with the quantification of micropollutants in whole-body extracts of gammarids that were collected at sites along the studied rivers.

## 2. Material and Methods

### 2.1. Study Area and Design

**Figure 1:**
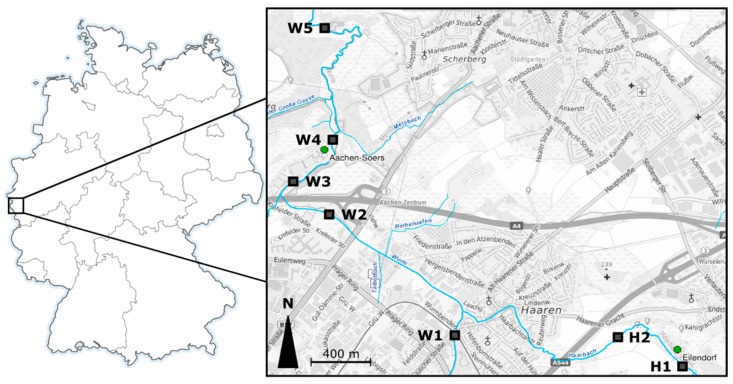
Study area with sampling sites along the River Wurm (W1–W5) and the River Haarbach (H1 and H2). © Data source ELWAS-WEB 2019, dl-de/by-2-0 (www.govdata.de/dl-de/by-2-0) https://www.elwasweb.nrw.de.

The River Wurm has a length of 53 km and has its source in forests south-west of Aachen, Germany. According to a recent report by the Ministry for Environment, Agriculture, Conservation, and Consumer Protection of the state of North Rhine-Westphalia, the catchment area of the River Wurm is dominated by agriculture (42.3%) and settlements or industry (30.6%), while only a small fraction is covered by grassland (13.6%) and forests (9.7%) [39]. Consequently, the Wurm is predominantly influenced by agriculture as well as by urban run-off, traffic, industry, and treated wastewater [40]. The River Haarbach is a tributary of the River Wurm, has a length of 13.5 km, and flows into the River Wurm approximately 2 km upstream of the WWTP Aachen-Soers. The WWTPs Aachen-Soers and the Eilendorf have population equivalents of 458,000 and 87,000, which translate into a total volume of sewage of 98,000,000 L/day and 13,000 L/day, respectively. As a consequence, the Haarbach consists of at least 50% treated effluent, while the proportion of treated wastewater is more than 70% of the River Wurm downstream of the WWTP Aachen-Soers; thus, sewage dominates the water system quantitatively and in terms of substance loads [41]. Due to their location upstream of the WWTPs, the sampling sites W1 and H1 were considered as reference sites to determine the influence of the WWTPs Aachen-Soers and Eilendorf. The study area with the seven sampling sites is shown in Figure 1.

### 2.2. Feeding Rate Inhibition

An in situ feeding rate inhibition test was used to determine the influence of discharged wastewater on the shredder performance of the freshwater amphipod *G. pulex*. Prior to the experiments, cages were built, leaf discs were prepared, and test organisms were collected (for detailed information see Supplementary Information (Supplementary Materials, Section 1). On the first day of the experiment, equally-sized *G. pulex* of approximately 1.5 cm were placed individually in the numbered cages, which contained two leaf discs. A total number of 25 cages were deployed at each site. In order to consider biotic and abiotic factors, which can alter the leaf weight, five cages contained leaf discs only. In order to reduce the uncertainty and variability of data sets, seven independent experiments lasting one week each were conducted in October and December 2015, January and July 2016, and July, August, and October 2017. Experiments set up in May 2017 and April 2018 were excluded due to bad weather and water flow conditions during which cages were lost.

From July 2016 on, the temperature was logged at every sampling site in order to evaluate possible effects on feeding rate caused by changes in water temperature. In 2017, three additional sampling sites (H1, H2, and W1) were added as experimental sites in order to obtain information on prior influences of tributaries on the River Wurm.

#### 2.2.1. Calculation of Feeding Rate

The feeding rate *C*, expressed as the dry weight of gammarids per dry weight of leaves per day, was calculated according to Maltby et al. [42]:

(1) $$C=\frac{\left(L_{1}\cdot C_{L}\right)-L_{2}}{W\cdot T}$$

*L*_1_ is the initial dry weight of the leaf disc in mg, *L*_2_ is the dry weight of the remaining leaf material in mg, *W* is the dry weight of the test organism in mg, *C_L_* is the correction factor and *T* is the time of deployment (7 days). The latter was calculated using

(2) $$C_{L}=\frac{\sum_{i}C_{2,i}/C_{1,i}}{N}$$

where *C*_1_ represents the dry weight of the control leaves in mg before deployment and *C*_2_ stands for the dry weight of the control leaves in mg after the test. *N* is the total amount of control leaves at each site.

#### 2.2.2. Statistical Analysis

Statistical analysis was performed with SigmaPlot (Version 12, Systat Software Inc., San Jose, CA, USA). Data were tested for normality using the Shapiro-Wilks test and for homogeneity of variance using Levene’s test. If samples were normally distributed and homogeneous in variance, a one-Way ANOVA was performed with the Bonferroni correction. Otherwise, the Kruskal-Wallis test or a two-way ANOVA on ranks was used (for detailed information see Supplementary Materials, Section 1.5).

### 2.3. Biota Analyses

A total of 60 analytes ranging in hydrophobicity from log *K_OW_* −0.2 to log *K_OW_* 5.5 were selected for internal concentration analysis based on their occurrence in water samples and sediments. In order to quantify organic micropollutants, 900 mg of gammarids from sampling sites W3, W4, and W5 (February 2016) and W3, W4, W5, and H1 (May 2017), respectively, were collected. Extracts were prepared according to a multi-target screening method developed by Inostroza et al. [43] (for detailed information, see Supplementary Materials, Section 2.2). The extracts were analysed by liquid chromatography coupled with high-resolution mass spectrometry (LC-HRMS, Thermo Fisher Scientific, Waltham, MA, USA; detailed information in Supplementary Materials, Section 2.1). Due to a lack of organisms at W1, W2, and H2, organic micropollutants were not measured in the biota from these sampling sites.

#### Toxic Pressure

To translate chemical concentrations into ecotoxicologically relevant and comparable values, the inherent toxicity, expressed in toxic units (TUs), was determined for each compound that was quantified by chemical analysis. TU was calculated by dividing the measured concentration by the acute EC_50_ (48 h) for either *G. pulex*, or, if no effect data was available, *Daphnia magna* (Supplementary Materials, Table S8). However, since EC_50_ values are almost exclusively based on water concentrations instead of internal concentrations, the measured internal concentrations were converted into their freely dissolved forms (*C^fd^*) (μg/L) of the respective micropollutant (Equation 3) [44]. To estimate the *C^fd^*, the total measured concentration (*C^t,G^*) in the gammarids was divided by the lipid content (*f_lipid_*) and the *K_OW_* was used as a surrogate for *K_lipid_*. Since the lipid content was not determined for the used gammarids due to the limited sample amount, a lipid fraction of 1.34% (*w/w*) was assumed [36,45].

(3) $$C^{fd}=\frac{C^{t,G}}{f_{lipid}\cdot K_{OW}}$$

In order to determine the mixture toxicity of all compounds that were detected in the gammarids, the TUs were summed up to sumTU (Equation 4), which is based on the assumption of toxicity additivity [46]. If the threshold value of −3.0 was exceeded by the sumTU, chronic effects cannot be excluded [47].

(4) $$sumTU=log\sum \left(\frac{C_{i}^{fd}}{EC_{50.i}}\right)$$

## 3. Results

### 3.1. Feeding Rate Inhibition

**Figure 2:**
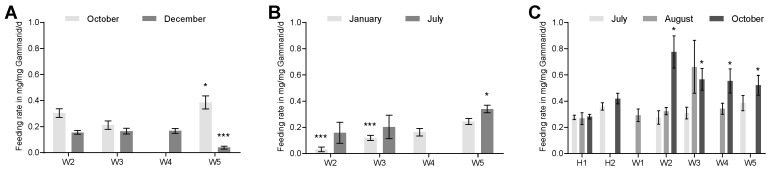
Feeding rates for the seven independent experiments conducted in 2015 (**A**), 2016 (**B**), and 2017 (**C**). The bars in (**A**–**C**) show the mean feeding rate calculated for each period at the respective sampling site, while the error bars depict the standard error of mean (SEM) of each measurement. The sampling sites were compared to each other using the Bonferroni correction (*** *p* < 0.001) for one-way ANOVA; otherwise, ANOVA on ranks was used (* *p* < 0.05). See raw data in Supplementary Materials, Tables S1–S4.

In order to evaluate the effects of wastewater on the shredder performance of *G. pulex*, the feeding rates at all sampling sites were compared to each other. In 2015, no trend in feeding rate alteration was observed among the different sampling sites (Figure 2A). In December, the feeding rate 2.5 km downstream of the WWTP Aachen-Soers was significantly reduced compared to that upstream (*p* < 0.001). The results from October showed the opposite, with the feeding rate at W5 significantly increased compared to W3 (*p* < 0.05). For January and July 2016, an increase in the feeding rate downstream of the WWTP Aachen-Soers can be observed (Figure 2B). In January, this effect was significant for W5 compared to both upstream sampling sites and for W4 compared to W2 (*p* < 0.05). In July, both sampling sites upstream from the WWTP Aachen-Soers were significantly different from sampling site W4 (*p* < 0.001). In summer 2017, no significant differences among the sites were observed (Figure 2C). In October 2017, no significant difference was detected, except for all Wurm sampling sites having significantly greater feeding rates compared to H1 (*p* < 0.05) (Figure 2C). Furthermore, there was a slight increase in the feeding rate downstream of the WWTP Eilendorf compared to upstream, which was not significant. Feeding rates in October 2017 were greater compared to those in other months and years. By comparing the feeding rates up- and downstream of the WWTP Aachen-Soers, no general trend of increased or decreased feeding rate could be found. However, in three out of four cases with a significant difference between sampling sites up- and downstream of the WWTP Aachen-Soers, an increase in feeding rate was detected. Additionally, no seasonal impact on the feeding rate was observed. No influence of temperature could be detected during a sampling campaign as some data loggers were lost during the experiments (Supplementary Materials, Table S5). For example, during measurement in October 2017, a slight increase in feeding rate was observed at the Haarbach with a temperature increase of 3.2 °C. However, there was no increase in the feeding rate along the Wurm from sampling site W3 (12 °C) to W4 (16.8 °C) or W5 (15.9 °C).

### 3.2. Biota Analyses

**Figure 3:**
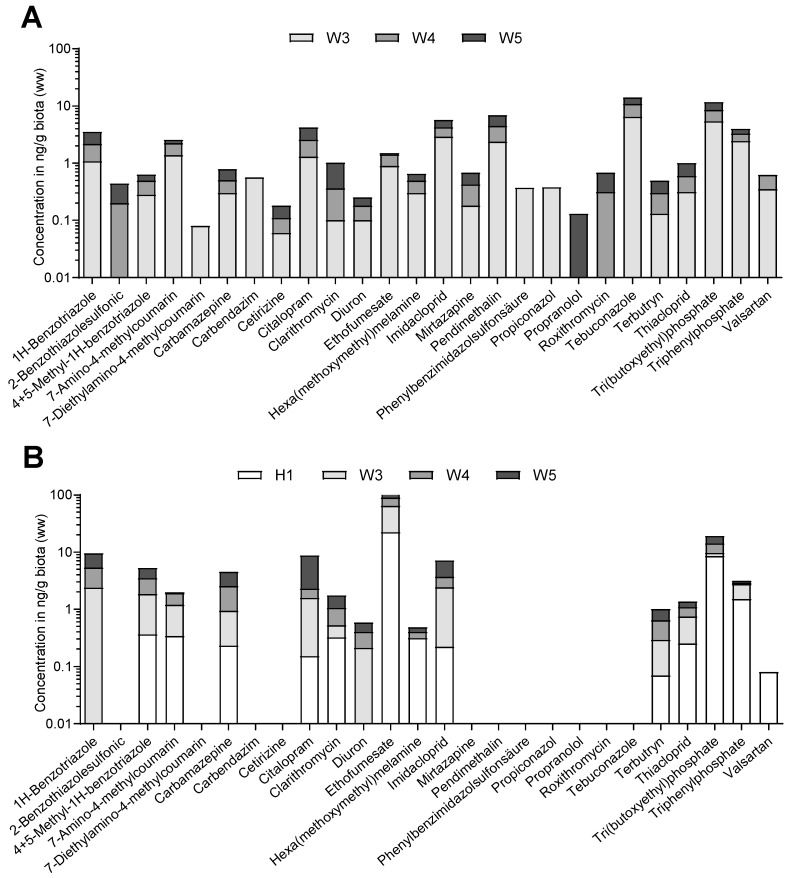
Results of biota analyses for sampling sites at which a sufficient amount of gammarids were obtained. Concentrations of quantified substances are shown for the sampling performed in February 2016 (**A**) and May 2017 (**B**).

The following graphs depict the internal concentrations in ng/g biota (wet weight) of substances that were detected in the extracts of gammarids collected at the sampling sites H1, W3, W4, and W5 (Figure 3A,B). For gammarids sampled in February 2016, 26 out of 60 substances belonging to different classes, such as pharmaceuticals, flame retardants, and pesticides, were quantified at similar concentrations in the range of 1 to 10 ng/g. The gammarid extracts at site W3 were dominated by the fungicide tebuconazole (6.5 ng/g), the flame retardant tri(butoxyethyl)phosphate (TBEP) (5.4 ng/g), and imidacloprid (2.9 ng/g), a worldwide used insecticide (Figure 3A). Moreover, the herbicide pendimethalin and the flame retardant triphenyl phosphate (TPP) both showed internal concentrations of 2.4 ng/g. 1H-Benzotriazole, 7-amino-4-methylcoumarin, citalopram, and ethofumesate were detected at concentrations around 1 ng/g. Further downstream at sampling site W4 (downstream WWTP), tebuconazole still dominated with a concentration of 4.4 ng/g, again followed by TBEP with 3.1 ng/g. Unlike W3, imidacloprid at W4 was detected at a lower tissue concentration than pendimethalin (2.1 ng/g). Additionally, TPP decreased in concentration from W3 to W4, while most of the other compounds remained relatively stable in concentration. At sampling site W5, the detected internal concentrations were very similar to those measured at W4. However, tebuconazole further decreased in concentration (3.2 ng/g), while TBEP and pendimethalin showed a slight increase, reaching 3.2 ng/g and 2.4 ng/g, respectively. In total, the highest sum concentration was measured for sampling site W3 (28 ng/g), while gammarids at W4 and W5 showed lower internal concentrations around 17 ng/g (Figure 3).

The direct comparison of the two sampling periods indicates a difference in the composition of quantified substances as well as in their pattern of occurrence. In the samples from May 2017 (Figure 3B), the predominantly present substances changed from being tebuconazole, TBEP, and imidacloprid to being ethofumesate, with 427 ng/g at W3, and TBEP, with a concentration of 8.5 ng/g at H1. Furthermore, the biocides pendimethalin and tebuconazole were not quantified. For Haarbach (H1), the majority of substances had internal concentrations lower than those in samples from the River Wurm. The few exceptions were TBEP, TPP, and hexa(methoxymethyl)melamine.

**Figure 4:**
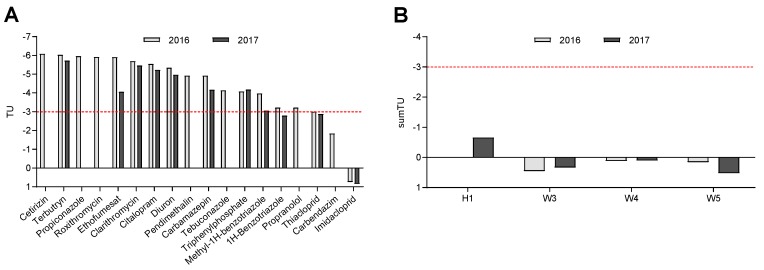
Toxic units (TUs) for single substances (**A**) and summarized TUs for each sampling site (**B**) for 2016 and 2017. For TUs above a value of −3.0 (below the dashed red line), chronic effects can be expected.

Based on the internal concentrations that were converted to freely dissolved concentrations (Supplementary Materials, Table S9), TUs and sumTUs were calculated. The TUs ranged −6.08–0.74 in 2016 and −5.72–0.84 in 2017 (Figure 4). The threshold value of −3.0, at which chronic effects can be expected, was exceeded for imidacloprid, carbendazim, thiacloprid, and 1H-benzotriazole, with imidacloprid being the substance contributing most to toxicity. The sumTUs determined for the sampling sites all exceeded the threshold value, ranging from 0.11 at W3 to 0.45 at W4 in 2016 and −0.66 at H1 to 0.52 at W5 in 2017 (Supplementary Materials, Table S10).

## 4. Discussion

The feeding rate of gammarids has been repeatedly demonstrated to be a sensitive and robust ecotoxicological endpoint upon which to determine the impact of wastewater on detritivorous macroinvertebrates. Previously conducted studies, which investigated the influence of wastewater on *Gammarus fossarum*, observed a reduction in feeding rates up to 70% under laboratory conditions [48]. These results were in accordance with feeding rates obtained from two different in situ studies using *G. fossarum*, during which a reduction of 80% and 90% was measured at 500 m and 50–150 m downstream of a WWTP, respectively [48,49]. Another study by Maltby et al. (2002) used *G. pulex* to monitor the water quality downstream of several different point source discharges [19]. At deployment sites that were influenced by released wastewater, a feeding rate inhibition ranging from approximately 20 to up to 90% was measured by Maltby et al. (2002). Based on these findings, the results of the present study, which detected no significant overall reduction in feeding rate for the sites downstream of the WWTP, were contrary to expectations. There were trends of an increased feeding rate downstream of the WWTP Aachen-Soers compared to the feeding rate upstream. One of the factors that might explain the differences between previously conducted studies and the present study is that different populations of gammarids that were used. These differences in sensitivity were also highlighted by Chaumot et al. (2015) [50], who stated that the variability in sensitivity to toxicants can occur between related individuals, between populations, and sibling species, which could be the case here. This interpopulation variability in sensitivity was also found by Crane et al. (1991), who tested the influence of water quality, test population, and test operator on the feeding rate of gammarids [51].

On the other hand, or even in addition to possible differences in sensitivity among populations, chemical concentrations released by the WWTP Aachen-Soers simply might not have exceeded the concentrations at which acute effects would occur [52,53]. A comparison of the corresponding chemical analyses for this study and a study by Bundschuh and Schulz [48] reveals lower chemical concentrations in the secondary clarifier of the WWTP Aachen-Soers. While in samples from the WWTP Wüeri (60% wastewater fraction), average concentrations of, for example, 1340 ng/L of 4-acetamidoantipyrine and 178 ng/L of isoproturon were measured [5], concentrations of 66 ng/L of 4-acetamidoantipyrine and 20 ng/L of isoproturon were measured at the WWTP Aachen-Soers effluent (70 to 90% wastewater fraction) (data not shown). Due to very low concentrations at which micropollutants are discharged into the streams, chronic effects—not covered with short-term feeding rate assays—are more likely to occur than acute effects.

Since benthic organisms such as gammarids continuously take up substances from the water column and sediments, they act as a passive sampler for bioavailable substances [35]. Thus, they can be used as a time-integrative tool in water quality assessment by way of analysing the substances that are accumulated in the tissues. The internal concentrations that were measured in gammarids from the River Wurm were in accordance with concentrations that were found in gammarids from the River Holtemme [44]. Both streams are substantially influenced by treated wastewater and thus contain a typical set of substances, such as 1H-benzotriazole, 5-methyl-1H-benzotriazole, carbamazepine, and tebuconazole. Moreover, a comparison to two other studies shows that gammarids in the River Danube exhibit lower internal concentrations than organisms from the River Wurm due to a lower wastewater fraction and a higher dilution, whereas gammarids in the agricultural streams Sauerbach and Getel (Germany) contained to up to one thousand-fold higher concentrations, taking the extreme case of the herbicide pendimethalin [43,54]. By combining internal concentrations of substances extracted from gammarids and the calculation of toxic units, toxic pressure on the gammarids and the potential for the occurrence of chronic effects can be estimated. Due to their low effect concentrations (Supplementary Materials, Table S8), the insecticides imidacloprid and thiacloprid, and the fungicide carbendazim, as well as the corrosion inhibitor 1H-benzotriazole, were identified as contributing most to the overall toxic pressure by being present at concentrations at which chronic effects can occur (TU ≥ −3.0) [47,55]. With imidacloprid being the most influential driver of toxicity, the sumTUs for all sampling sites were determined to be above −3.0. However, if all calculations were based on *D. magna* data, the overall toxic pressure would be underestimated, as *D. magna* is less sensitive to carbamazepin, imidacloprid, and thiacloprid (Supplementary Materials, Table S11). While for both years the toxic pressure was approximately the same at sampling site W4, there were many differences for the sites W3 and W5 between February 2016 and May 2017. A possible explanation could be the land-use of the surrounding area. W5 is predominantly influenced by agricultural run-off and thus might receive high inputs of pesticides during their application periods in spring and early summer, which fits well with the observation of elevated TUs in May compared to February. By contrast, sampling site W3 is much more impacted by urban run-off due to its position at the outlet of a storm water overflow tank, which releases untreated wastewater during events of heavy rainfall. In the present study, gammarids collected from downstream of the storm water overflow tank in February accumulated more substances in greater concentrations than gammarids collected at other sampling sites (Supplementary Materials, Table S6), which might be explained by untreated wastewater releases that are likely to occur during the winter and early spring.

A consequence of long-term exposure to the substances that were found in considerably high internal concentrations in gammarids is that several different endpoints can be affected. It has been demonstrated by De Lange et al. (2006) and Dietrich et al. (2010) that sublethal concentrations of pharmaceuticals, especially when they are present as a mixture, can alter the behavior, ventilation rates, locomotion, and molting behavior of gammarids [53,56]. Due to the key role that gammarids play for leaf litter breakdown, these general signs of stress leading to an increase in mortality can severely impact the benthic community and the whole ecosystem [57]. *G. pulex* populations inhabiting freshwater systems affected by man-made chemicals or chemicals from agricultural activities and WWTPs can alter population genetic patterns. These genetic changes may lead to changes in ecological functioning and ultimately fitness [54].

## 5. Conclusions

In this study, it was not possible to observe clear acute effects with a feeding inhibition assay, as the chemical load was too low. Furthermore, it was observed that species differences can have a high impact on behaviour and have to be taken into account, in addition to seasonal variations and the accompanying chemical composition. However, by supplementation of the acute feeding rate inhibition test with internal concentration analysis, it was possible to gain information on the chronic effect potential of the river system, which was not depicted by the acute in situ experiment. A further approach could be the combination of feeding experiments with a final measurement of the internal concentration of gammarids to be able to directly link the internal concentrations in the tissues with the measured feeding rates during the experimental time. Additional laboratory experiments with *G. pulex* with the substances that were identified as drivers of toxicity should be conducted to gain more information on the toxicity profile of the investigated area.

## Supplementary Materials

The following are available online at https://www.mdpi.com/1660-4601/16/5/883/s1. Table S1: Measured feeding rates in mg/mg gammarid/day per cage (raw data) for 2015, Table S2: Measured feeding rates in mg/mg gammarid/day per cage (raw data) for 2016, Table S3: Measured feeding rates in mg/mg gammarid/day per cage (raw data) for 2017, Table S4: Measured feeding rates in mg/mg gammarid/day per cage (raw data) for 2017, Table S5: Measured temperatures (°C) at the sampling sites, Table S6: Determined internal concentrations in ng/g wet weight of gammarid tissue, Table S7: LogK_ow_ of substances detected in gammarid tissues. For substances where no experimental value was available, the logK_ow_ was estimated using EPIsuite, Table S8: Median acute effect concentrations in μg/L for *Gammarus pulex* and *Daphnia magna* after 48 h. EC_50_ values without reference (*) were taken from the Indicate Software Version 1.0.0 (UFZ Leipzig), Table S9: Estimated freely dissolved water concentrations (C^fd^) in µg/L, Table S10: Toxic Units for each compound and sumTUs for sampling sites. A value above -3.0 indicates the occurrence of chronic effects. Calculations for carbamazepin, imidacloprid and thiacloprid are based on EC_50_ values from *G. pulex*, Table S11: Toxic Units for each compound and sumTUs for sampling sites. A value above -3.0 indicates the occurrence of chronic effects. All calculations are based on EC_50_ values from *D. magna*.
