# Supplementary material for: Combination of In Situ Feeding Rate Experiments and Chemical Body Burden Analysis to Assess the Influence of Micropollutants in Wastewater on Gammarus pulex
Source: Int J Environ Res Public Health. 2019 Mar 11;16(5):883. doi: 10.3390/ijerph16050883 (PMC6427342; doi:10.3390/ijerph16050883)
Supplement: Supplementary file 1 [file ijerph-16-00883-s001.zip › ijerph-434932-suppl xml.pdf]

Supplementary Information

Combination of in situ feeding rate experiments and chemical body burden analysis to assess the influence of micropollutants in wastewater on *Gammarus pulex*

Sarah Könemann[1,2,*,‡], Yvonne Müller[1,‡], Daniel Tschentscher[1], Martin Krauss[3], Pedro A. Inostroza[3,4], Ira Brückner[5], Johannes Pinnekamp[6], Sabrina Schiwy[1], Henner Hollert[1,*]

1 Department of Ecosystem Analysis, Institute for Environmental Research, RWTH Aachen University, Worringerweg 1, 52074 Aachen, Germany

2 Current address: Department Environmental Toxicology, Eawag, Überlandstrasse 133, 8600 Dübendorf, Switzerland

3 Department Effect-Directed Analysis, Helmholtz Institute for Environmental Research - UFZ, Permoserstraße 15, 04318 Leipzig, Germany

4 Current address: Department of Biological and Environmental Sciences, University of Gothenburg, PO BOX 461, 40530 Gothenburg, Sweden

5 Waterboard Eifel-Rur, Eisenbahnstr. 5, 52353 Düren, Germany

6 Institute of Environmental Engineering, RWTH Aachen University, Mies-van-der-Rohe-Str. 1, 52074 Aachen, Germany

‡The authors contributed equally to the preparation of this manuscript.

*Correspondence authors: sarah.koenemann@rwth-aachen.de and Henner.Hollert@bio5.rwth-aachen.de

1 Feeding rate inhibition

1.1 Preparation of cages

High-density polyethylene (HDPE) boxes, which measured 5 cm in height and 3.6 cm in diameter were used to build suitable cages for enclosure of single specimen. To ensure steady perfusion, holes of approximately 2 cm were cut in lid and bottom of the boxes, which then were covered with gauze of 900 mm mesh size. Cages were numbered and a number of 25 was fixed to a wire, respectively. Before use, the cages were watered in tab water for 24 h.

1.2 Preparation of leaf discs

Senescent but undecomposed leaves of black alder (*Alnus glutinosa* L. Gaertn.) were collected before leaf fall in autumn and stored at -20°C until used. Leaf discs were prepared on the basis of Bundschuh and Schulz (2011) [1]. Two weeks before each experiment, approximately 50 leaves were thawed and conditioned in aerated artificial pond water (APW) (prepared according to Naylor et al., (1989) [2]) at 15°C ± 1°C. To facilitate and accelerate the establishment of a microbial community on the leaves, naturally conditioned alder leaves from a small creek were added. After 14 d incubation time, 200 leaf discs measuring 2 cm in diameter were cut with a cork borer. Then, the leaf discs were randomised and distributed in pairs to small, pre-weighed and numbered aluminium bowls. The discs were dried at 60°C to constant weight for approximately 24 h and weighed to the nearest 0.01 mg (UMX Comparator, Mettler-Toledo GmbH, Switzerland). The day before the test, the leaf discs were transferred to the numbered cages and were soaked in APW for 24 h.

1.3 Test organisms

One week before each experiment, the test organisms were obtained from a small creek near Aachen, Germany (50°511 N; 6°81 E). The collected gammarids were visually checked for acanthocephalan parasites. Since infected specimen can show abnormal behaviour, these individuals were not used for

the experiment. Afterwards, the remaining organisms were kept in an aerated APW at 15°C ± 1°C and a light-dark cycle of 16:8 h. Preconditioned alder leaves were provided as food.

1.4 Data

Table S1: Measured feeding rates in mg/mg gammarid/day per cage (raw data) for 2015.

October 2015			December 2015			
**W2**	**W3**	**W5**	**W2**	**W3**	**W4**	**W5**
0.318	0.076	0.4988	0.122	0.127	0.157	0.063
0.340	0.281	0.4616	0.130	0.133	0.077	0.045
0.202	0.483	0.3097	-0.028	0.073	0.166	0.125
0.202	0.017	0.4575	0.130	0.125	0.230	0.060
0.364	0.428	0.3267	0.269	0.180	0.252	0.128
0.326	0.256	0.2571	0.167	0.170	0.139	0.048
0.389	0.269	0.0414	0.133	0.116	0.133	0.090
0.410	0.181	0.2597	0.150	0.122	0.113	0.012
0.584	0.128	0.2979	0.218	0.002	0.138	0.122
0.358	0.021	0.2196	0.184	0.172	0.105	0.027
0.223	0.025	0.2945	0.187	0.179	0.156	-0.020
0.236	0.175	0.4688	0.197	0.087	0.208	0.002
0.182	0.158	0.3068	0.086	0.215	0.358	-0.027
0.150	0.493	0.6128	0.204	0.131	0.119	0.014
0.750	0.001	1.0825	0.204	0.127		0.013
0.262	0.239	0.2645	0.136	0.165		0.100
0.270	0.242	0.5655	0.112	0.395		0.002
0.170	0.300	0.2348	0.126	0.445		-0.029
0.135	0.237	0.3838	0.130	0.159		0.020
0.224	0.232	0.4988	0.256	0.185		-0.007

Table S2: Measured feeding rates in mg/mg gammarid/day per cage (raw data) for 2016.

January 2016			July 2016			
**W2**	**W3**	**W4**	**W5**	**W2**	**W3**	**W5**
-0.098	0.070	0.212	0.246	0.156	0.239	0.261
-0.029	0.126	0.151	0.220	0.075	0.301	0.240
-0.016	0.111	0.120	0.270	0.174	0.185	0.402
0.148	0.065	0.215	0.288	0.123	0.014	0.549
-0.116	0.016	0.176	0.412	0.137	0.164	0.237
0.086	0.063	0.210	0.354	0.278	0.125	0.405
0.114	0.292	0.405	0.111	0.121	0.162	0.218
0.125	0.103	-0.055	-0.074	0.201	0.244	0.335
0.040	0.077	0.163	0.147		0.252	0.355
0.106	0.202	0.177	0.222		0.195	0.103
0.053	0.214	0.126	0.316		0.208	0.425
0.012	0.065	0.290	0.293		0.063	0.343
0.119	-0.051	0.149	0.283		0.160	0.393
-0.007	0.277	0.301	0.245		0.269	0.387
-0.002	0.103	0.208	0.231		0.134	0.440
-0.062	0.130	0.171	0.311		0.425	
0.103	0.121	-0.195	0.217		0.307	
-0.026	0.194	0.276	0.221		0.239	
0.047	0.035	0.182	0.310		0.301	
	0.176	-0.023	0.269		0.185	

Table S3: Measured feeding rates in mg/mg gammarid/day per cage (raw data) for 2017.

July 2017					August 2017				
**H1**	**H2**	**W2**	**W3**	**W5**	**H1**	**W1**	**W2**	**W3**	**W4**
0.283	0.509	0.275	0.546	0.793	0.328	0.334	0.169	0.588	0.369
0.327	0.554	0.212	0.403	0.460	0.590	-0.088	0.204	0.413	0.384
0.218	0.250	0.036	0.481	0.454	0.251	0.319	0.370	0.241	0.291
0.311	0.333	0.347	-0.255	-0.135	0.242	0.362	0.368	0.428	0.277
0.185	0.272	0.627	0.108	0.536	0.163	0.388	0.263	0.042	0.423
0.152	0.517	0.660	0.275	0.232	0.463	0.742	0.365	0.509	0.498
0.298	0.477	0.396	0.438	0.319	0.528	0.049	0.203	0.347	0.156
0.421	0.322	-0.233	0.481	0.526	0.287	0.217	0.377	0.346	
0.316	0.287	0.200	0.345	0.802	0.461	0.218	0.488	0.283	
0.358	0.213	0.328	0.226	0.448	0.283	0.253	0.353	3.694	
0.338	0.242	0.391	0.329	0.254	0.247	0.330	0.378	0.685	
0.398	0.397	0.192	0.371	0.403	0.255	0.540	0.352	0.570	
0.211	0.155	0.005	0.150	0.200	-0.001	0.266		0.583	
0.247	0.410	0.326	0.377	0.364	0.052	0.251		0.300	
0.216	0.266	0.376	0.328	0.607	0.125	0.195		1.402	
0.270	0.548	0.216	0.134	0.132	-0.005			0.238	
0.210	0.272	0.317	0.330	0.166				0.579	
0.285	0.543		0.501						
0.201	0.328								
	0.309								

Table S4: Measured feeding rates in mg/mg gammarid/day per cage (raw data) for 2017.

October 2017					
**H1**	**H2**	**W2**	**W3**	**W4**	**W5**
0.245	0.567	0.887	0.465	0.326	0.480
0.288	0.620	0.659	1.496	0.451	0.428
0.416	0.689	0.837	0.711	0.432	0.610
0.399	0.606	0.020	0.371	0.530	0.823
0.205	0.672	1.741	0.528	0.674	0.531
0.263	0.255	1.014	0.434	0.462	0.572
0.184	0.220	0.441	0.484	0.234	0.656
0.216	0.494	0.808	0.781	0.641	0.288
0.319	0.345	0.588	0.682	0.542	-0.166
0.386	0.299	0.127	0.479	1.599	0.630
0.163	0.211	0.854	0.473	0.170	0.611
0.216	0.385	0.902	0.442	0.358	0.789
0.323	0.369	1.204	0.460	0.796	
0.299	0.195		0.133	0.545	
0.217	0.305				
0.200	0.445				
0.311	0.457				
0.414					
0.365					
0.203					

Table S5: Measured temperatures (°C) at the sampling sites.

	July 2016	July 2017	August 2017	October 2017
**W1**			18.4	
**H1**		16.8		10.5
**H2**		19.0		13.7
**W2**	18.8	18.2		
**W3**	18.3	17.7	18.0	12.0
**W4**				16.8
**W5**				15.9

1.5 Statistical Analysis

October 2015

One Way Analysis of Variance

Normality Test (Shapiro-Wilk) Failed (P < 0,050)

Test execution ended by user request, ANOVA on Ranks begun

Kruskal-Wallis One Way Analysis of Variance on Ranks

Group	N	Missing	Median	25%	75%
W2	19	0	0,27	0,202	0,364
W3	19	0	0,237	0,0765	0,281
W5	18	0	0,308	0,259	0,476

H = 8,723 with 2 degrees of freedom. (P = 0,013)

The differences in the median values among the treatment groups are greater than would be expected by chance; there is a statistically significant difference (P = 0,013)

To isolate the group or groups that differ from the others use a multiple comparison procedure.

All Pairwise Multiple Comparison Procedures (Dunn's Method):

Comparison	Diff of Ranks	Q	P<0,05
W5 vs W3	15,74	2,934	Yes
W5 vs W2	6,529	1,217	No
W2 vs W3	9,211	1,741	No

December 2015

One Way Analysis of Variance

Normality Test (Shapiro-Wilk) Passed (P = 0,150)

Equal Variance Test: Passed (P = 0,787)

Group Name	N	Missing	Mean	Std Dev	SEM
W2	16	0	0,156	0,0667	0,0167
W3	16	0	0,133	0,0506	0,0127
W4	10	0	0,151	0,0541	0,0171
W5	16	0	0,05	0,0511	0,0128

Source of Variation	DF	SS	MS	F	P
**Between Groups**	3	0,111	0,0369	11,672	<0,001
**Residual**	54	0,171	0,00316		
**Total**	57	0,281			

The differences in the mean values among the treatment groups are greater than would be expected by chance; there is a statistically significant difference (P = <0,001).
Power of performed test with alpha = 0,050: 0,999
All Pairwise Multiple Comparison Procedures (Bonferroni t-test):

Comparison	Diff of Means	t	P	P<0,050
W2 vs. W5	0,105	5,306	<0,001	Yes
W2 vs. W3	0,0228	1,148	1	No
W2 vs. W4	0,00448	0,198	1	Do Not Test
W4 vs. W5	0,101	4,456	<0,001	Yes
W4 vs. W3	0,0183	0,809	1	Do Not Test
W3 vs. W5	0,0827	4,159	<0,001	Yes

A result of "Do Not Test" occurs for a comparison when no significant difference is found between two means that enclose that comparison.

January 2016

One Way Analysis of Variance
Normality Test (Shapiro-Wilk) Failed (P < 0,050)
Test execution ended by user request, ANOVA on Ranks begun
Kruskal-Wallis One Way Analysis of Variance on Ranks

Group	N	Missing	Median	25%	75%
W2	19	0	0,0402	-0,0264	0,106
W3	20	0	0,107	0,065	0,189
W4	20	0	0,176	0,132	0,214
W5	20	0	0,257	0,22	0,306

H = 35,029 with 3 degrees of freedom. (P = <0,001)
The differences in the median values among the treatment groups are greater than would be expected by chance; there is a statistically significant difference (P = <0,001)
To isolate the group or groups that differ from the others use a multiple comparison procedure.
All Pairwise Multiple Comparison Procedures (Dunn's Method):

Comparison	Diff of Ranks	Q	P<0,05
W5 vs W2	42,018	5,715	Yes
W5 vs W3	26,4	3,638	Yes
W5 vs W4	15,25	2,101	No
W4 vs W2	26,768	3,641	Yes
W4 vs W3	11,15	1,536	No
W3 vs W2	15,618	2,124	No

July 2016

One Way Analysis of Variance
Normality Test (Shapiro-Wilk) Passed (P = 0,873)
Equal Variance Test: Passed (P = 0,241)

Group Name	N	Missing	Mean	Std Dev	SEM
W2	8	0	0,158	0,0614	0,0217
W3	17	0	0,203	0,0971	0,0235
W5	15	0	0,34	0,111	0,0286

Source of Variation	DF	SS	MS	F	P
**Between Groups**	2	0,225	0,113	11,924	<0,001
**Residual**	37	0,349	0,00944		
**Total**	39	0,574			

The differences in the mean values among the treatment groups are greater than would be expected by chance; there is a statistically significant difference (P = <0,001).

Power of performed test with alpha = 0,050: 0,991

All Pairwise Multiple Comparison Procedures (Bonferroni t-test):

Comparison	Diff of Means	t	P	P<0,050
**W5 vs. W2**	0,181	4,267	<0,001	Yes
**W5 vs. W3**	0,137	3,978	<0,001	Yes
**W3 vs. W2**	0,0446	1,071	0,874	No

July 2017

One Way Analysis of Variance

Normality Test (Shapiro-Wilk) Failed (P < 0,050)

Test execution ended by user request, ANOVA on Ranks begun

Kruskal-Wallis One Way Analysis of Variance on Ranks

Group	N	Missing	Median	25%	75%
H1	19	0	0,283	0,211	0,327
H2	20	0	0,325	0,268	0,501
W1	19	0	0,361	0,279	0,547
W2	17	0	0,317	0,196	0,383
W3	18	0	0,338	0,207	0,449
W5	17	0	0,403	0,216	0,531

H = 8,037 with 5 degrees of freedom. (P = 0,154)

The differences in the median values among the treatment groups are not great enough to exclude the possibility that the difference is due to random sampling variability; there is not a statistically significant difference (P = 0,154)

August 2017

One Way Analysis of Variance

Normality Test (Shapiro-Wilk) Failed (P < 0,050)

Test execution ended by user request, ANOVA on Ranks begun

Kruskal-Wallis One Way Analysis of Variance on Ranks

Group	N	Missing	Median	25%	75%

H1	16	0	0,253	0,135	0,428
W1	15	0	0,266	0,217	0,362
W2	12	0	0,359	0,219	0,375
W3	17	0	0,428	0,292	0,585
W4	7	0	0,369	0,277	0,423

H = 9,147 with 4 degrees of freedom. (P = 0,058)
The differences in the median values among the treatment groups are not great enough to exclude the possibility that the difference is due to random sampling variability; there is not a statistically significant difference (P = 0,058).

October 2017

One Way Analysis of Variance
Normality Test (Shapiro-Wilk) Failed (P < 0,050)
Test execution ended by user request, ANOVA on Ranks begun
Kruskal-Wallis One Way Analysis of Variance on Ranks

Group	N	Missing	Median	25%	75%
H1	20	0	0,276	0,208	0,354
H2	17	0	0,385	0,277	0,586
W2	13	0	0,837	0,515	0,958
W3	14	0	0,476	0,44	0,689
W4	14	0	0,496	0,35	0,649
W5	12	0	0,591	0,441	0,65

H = 29,066 with 5 degrees of freedom. (P = <0,001)
The differences in the median values among the treatment groups are greater than would be expected by chance; there is a statistically significant difference (P = <0,001)
To isolate the group or groups that differ from the others use a multiple comparison procedure.
All Pairwise Multiple Comparison Procedures (Dunn's Method):

Comparison	Diff of Ranks	Q	P<0,05
W2 vs H1	44,577	4,789	Yes
W2 vs H2	25,606	2,66	No
W2 vs W4	15,72	1,562	Do Not Test
W2 vs W3	12,505	1,243	Do Not Test
W2 vs W5	10,827	1,035	Do Not Test
W5 vs H1	33,75	3,538	Yes
W5 vs H2	14,779	1,5	Do Not Test
W5 vs W4	4,893	0,476	Do Not Test
W5 vs W3	1,679	0,163	Do Not Test
W3 vs H1	32,071	3,523	Yes
W3 vs H2	13,101	1,389	Do Not Test
W3 vs W4	3,214	0,326	Do Not Test
W4 vs H1	28,857	3,17	Yes
W4 vs H2	9,887	1,049	Do Not Test
H2 vs H1	18,971	2,201	No

2 Biota analyses

2.1 Extraction

Previous to the extraction all glass ware was cleaned with acetone (HPLC grade), ethyl acetate (HPLC grade), milli-Q water and methanol (gradient-grade). Then, 900 mg of each sample were thawed and transferred to glass centrifugation tubes (Carl Roth GmbH und Co. KG, Germany). 4 mL of a mixture of acetonitrile and milli-Q (1:1, v/v), and 1 mL of hexane were added. Using an Ultra-Turrax the samples were homogenised for 60 s and subsequently vortexed for another 60 s. For the first clean-up step, 800 mg of anhydrous MgSO4 and 200 mg of NaCl were added. To avoid agglomeration the mixture was vortexed for 1 min straightaway and centrifuged for 5 min at 4000 g. Afterwards, the hexane phase was removed with a glass pipette and stored in a cleaned amber vial at -20 °C. In order to improve analytical performance a dispersive SPE (dSPE) was conducted as second clean-up step. Therefore, the acetonitrile supernatant was allocated to glass centrifugation tubes containing 50 mg of primary secondary amine (PSA) and 400 mg of anhydrous MgSO4. The mixture was vortexed for 60 s and centrifuged for 5 min at 4000 g. The supernatant was transferred to a cleaned amber vial and MgSO4/PSA residue was extracted for a second time by adding 2 mL of acetonitrile and centrifuging again. Then, the combined supernatants were dried under a N2-stream at room temperature. Dry samples were reconstituted in 500 µL of MeOH, filtered (PTFE syringe filter, 0.45 µm pores, Chromafil) into an amber vial and stored at -20 °C.

2.2 Chemical analysis

For LC-HRMS analysis, a Thermo Ultimate 3000 system coupled to a Thermo QExactive Plus MS was used. The chromatographic separation was done on a Kinetex C18 EVO column (50 x 2.1 mm, 2.6 µm particle size) using a gradient elution with 0.1% of formic acid (eluent A) and methanol containing 0.1% of formic acid (eluent B) at a flow rate of 300 µL/min. After 1 min of 5% B, the fraction of B was linearly increased to 100% within 12 min and 100% B were kept for 11 min. The eluent flow was diverted to waste and the column was rinsed for 2 min using a mixture of isopropanol + acetone 50:50 / eluent B / eluent A (85% / 10% / 5%) to remove hydrophobic matrix constituents from the column. Finally, the column was re-equilibrated to initial conditions for 5.7 min. The injection volume was 5 µL and the column was operated at 40 °C. The heated ESI source and the transfer capillary were both operated at 300 °C, the spray voltage was 3.8 kV (positive mode) or 3.5 kV (negative mode), the sheath gas flow rate was 45 a.u. and the auxiliary gas flow rate 1 a.u. Separate runs were conducted in positive and negative ion mode combining a full scan experiment (100-1000 m/z) at a nominal resolving power of 70000 (referenced to m/z 200) and data-independent MS/MS experiments at a nominal resolving power of 35000. For the latter, they acquired the data using broad isolation windows of about 50 mu (i.e., m/z ranges 97-147, 144-194, 191-241, 238-288, 285-335, 332-382, 379-429, 426-476) and 280 mu (i.e., m/z ranges 460-740, 730-1010), respectively.

2.3 Data

Table S6: Determined internal concentrations in ng/g wet weight of gammarid tissue.

Substance	H1	W3		W4		W5	
	2017	2016	2017	2016	2017	2016	2017
1H-Benzotriazole	< LOQ	1.07	2.36	1.10	3.01	1.36	4.11
2-Benzothiazolsulfonic acid	< LOQ	0.25	< LOQ	0.20	< LOQ	0.24	< LOQ

4+5-Methyl-1H-benzotriazole	0.36	0.28	1.48	0.21	1.66	0.14	1.76
7-Amino-4-methylcoumarin	0.34	1.38	0.85	0.84	0.72	0.35	0.07
7-Diethylamino-4-methylcoumarin	< LOQ	0.08	< LOQ	< LOQ	< LOQ	< LOQ	< LOQ
Carbamazepine	0.23	0.30	0.70	0.20	1.62	0.28	1.94
Carbendazim	< LOQ	0.56	< LOQ	< LOQ	< LOQ	< LOQ	< LOQ
Cetirizin	< LOQ	0.06	< LOQ	0.05	< LOQ	0.07	< LOQ
Citalopram	0.15	1.29	1.43	1.27	0.70	1.64	6.45
Clarithromycin	0.32	0.10	0.20	0.26	0.53	0.66	0.68
Diuron	< LOQ	0.10	0.21	0.08	0.19	0.07	0.18
Ethofumesate	22.2	0.89	42.2	0.52	25.4	0.09	15.93
Hexa(methoxymethyl)melamine	0.31	0.30	< LOQ	0.19	0.09	0.16	0.08
Imidacloprid	0.22	2.90	2.17	1.33	1.28	1.46	3.42
Mirtazapine	< LOQ	0.18	< LOQ	0.24	< LOQ	0.26	< LOQ
Pendimethalin	< LOQ	2.37	< LOQ	2.10	< LOQ	2.41	< LOQ
Phenylbenzimidazole sulfonic acid	< LOQ	0.37	< LOQ	< LOQ	< LOQ	< LOQ	< LOQ
Propiconazole	< LOQ	0.38	< LOQ	< LOQ	< LOQ	< LOQ	< LOQ
Propranolol	< LOQ	< LOQ	< LOQ	0.00	< LOQ	0.13	< LOQ
Roxithromycin	< LOQ	< LOQ	< LOQ	0.31	< LOQ	0.37	< LOQ
Tebuconazole	< LOQ	6.45	< LOQ	4.38	< LOQ	3.24	< LOQ
Terbutryn	0.07	0.13	0.22	0.17	0.35	0.19	0.36
Thiacloprid	0.25	0.31	0.49	0.28	0.34	0.41	0.28
Tri(butoxyethyl) phosphate	8.52	5.38	1.08	3.11	4.51	3.17	4.95
Triphenyl phosphate	1.50	2.42	1.24	0.85	0.16	0.73	0.23
Valsartan	0.08	0.35	< LOQ	0.27	< LOQ	< LOQ	< LOQ
Denatonium	308	< LOQ	466	< LOQ	168	< LOQ	80.8

Table S7: LogK_{ow} of substances detected in gammarid tissues. For substances where no experimental value was available, the logK_{ow} was estimated using EPIsuite.

**Substance**	**LogK_{ow}**
1H-Benzotriazole	1.2
2-Benzothiazolsulfonic acid	-0.9
4+5-Methyl-1H-benzotriazole	1.7
7-Amino-4-methylcoumarin	1.1
7-Diethylamino-4-methylcoumarin	3.2
Carbamazepine	2.5
Carbendazim	1.5
Cetirizin	1.7
Citalopram	3.5
Clarithromycin	3.2
Diuron	2.7
Ethofumesate	2.9
Hexa(methoxymethyl)melamine	1.6
Imidacloprid	-0.4
Mirtazapine	2.9
Pendimethalin	5.2
Phenylbenzimidazole sulfonic acid	-0.2
Propiconazole	3.7
Propranolol	3.5
Roxithromycin	1.7
Tebuconazole	3.7
Terbutryn	3.7
Thiacloprid	1.3
Tri(butoxyethyl) phosphate	3.8
Triphenyl phosphate	4.6
Valsartan	4.0

Table S8: Median acute effect concentrations in µg/L for *Gammarus pulex* and *Daphnia magna* after 48 h. EC_{50} values without reference (*) were taken from the Indicate Software Version 1.0.0 (UFZ Leipzig).

Substances	EC_{50} *G. pulex*	EC_{50} *D. magna*	References
1H-Benzotriazole		15800	[3]
4+5-Methyl-1H-benzotriazole		8580	[3]
Carbamazepine	17000	70000	[3], [4]
Carbendazim		87.6	[5]
Cetirizin		330000	[6]
Citalopram		20000	[7]
Clarithromycin		25720 (24 h)	[8]
Diuron		8400	*
Ethofumesate		179000	*
Imidacloprid	20.59	97000	[3], [9]
Pendimethalin		280	[3]
Propiconazole		4900	*
Propranolol		5531	[10]
Roxithromycin		74300	[11]
Tebuconazole		2790	[3]
Terbutryn		2660	[3]
Thiacloprid	350	88000	[3], [9]
Triphenyl phosphate (TPP)		90	[12]
Valsartan		>580	[13]

Table S9: Estimated freely dissolved water concentrations (C^{fd}) in μg/L.

Substances	K_{OW}	H1	W3		W4		W5	
		2017	2016	2017	2016	2017	2016	2017
1H-Benzotriazole	27.5		2.9022	6.4030	2.9730	8.1602	3.6895	11.1479
2-Benzothiazolsulfonic acid	0.1		178.6740		146.2615		177.0533	
4+5-Methyl-1H-benzotriazole	51.3	0.5232	0.4066	2.1521	0.3088	2.4195	0.2069	2.5549
7-Amino-4-methylcoumarin	13.8	1.8199	7.4756	4.5798	4.5563	3.8745	1.8892	0.3810
7-Diethylamino-4-methylcoumarin	1659		0.0036					
Carbamazepine	282	0.0606	0.0791	0.1847	0.0538	0.4298	0.0736	0.5128
Carbendazim	33.1		1.2571					
Cetirizin	50.1		0.0918		0.0811		0.1026	
Citalopram	5495	0.0020	0.0175	0.0194	0.0172	0.0095	0.0223	0.0875
Clarithromycin	1445	0.0163	0.0053	0.0103	0.0137	0.0274	0.0341	0.0351
Diuron	478		0.0149	0.0332	0.0126	0.0298	0.0113	0.0274
Ethofumesate	501	3.3040	0.1332	6.2798	0.0773	3.7746	0.0127	2.3726
Hexa(methoxymethyl)melamine	40.7	0.5668	0.5465	0.0000	0.3562	0.1628	0.2890	0.1494
Imidacloprid	3.7	4.5119	58.3054	43.5088	26.8038	25.6321	29.2922	68.7519
Mirtazapine	1072		0.0127		0.0168		0.0180	
Pendimethalin	15135		0.0012		0.0010		0.0012	
Phenylbenzimidazole sulfonic acid	0.9		28.7772					
Propiconazole	5248		0.0054					
Propranolol	3020				2.9730		0.0031	
Roxithromycin	562				0.0409		0.0488	
Tebuconazole	5012		0.0960		0.0652		0.0483	
Terbutryn	5495	0.0010	0.0018	0.0030	0.0023	0.0047	0.0025	0.0049
Thiacloprid	213	0.0864	0.1084	0.1697	0.0973	0.1202	0.1441	0.0990
Tri(butoxyethyl) phosphate (TBEP)	589	1.0799	0.6823	0.1375	0.3944	0.5714	0.4015	0.6275
Triphenyl phosphate (TPP)	38905	0.0029	0.0046	0.0024	0.0016	0.0003	0.0014	0.0004
Valsartan	4467	0.0013	0.0059		0.0046			

Table S10: Toxic Units for each compound and sumTUs for sampling sites. A value above -3.0 indicates the occurrence of chronic effects. Calculations for carbamazepin, imidacloprid and thiacloprid are based on EC_{50} values from *G. pulex*.

Substances	H1	W3		W4		W5		logTU	
	2017	2016	2017	2016	2017	2016	2017	2016	2017
1H-Benzotriazole		1.84E-04	4.05E-04	1.88E-04	5.16E-04	2.34E-04	7.06E-04	**-3.22**	**-2.79**
2-Benzothiazolsulfonic acid									
4+5-Methyl-1H-benzotriazole	6.10E-05	4.74E-05	2.51E-04	3.60E-05	2.82E-04	2.41E-05	2.98E-04	**-3.97**	**-3.05**
7-Amino-4-methylcoumarin									
7-Diethylamino-4-methylcoumarin									
Carbamazepine	3.57E-06	4.66E-06	1.09E-05	3.17E-06	2.53E-05	4.33E-06	3.02E-05	**-4.92**	**-4.16**
Carbendazim		1.44E-02						**-1.84**	
Cetirizin		2.78E-07		2.46E-07		3.11E-07		**-6.08**	
Citalopram	1.01E-07	8.76E-07	9.72E-07	8.62E-07	4.76E-07	1.11E-06	4.38E-06	**-5.54**	**-5.23**
Clarithromycin	6.34E-07	2.05E-07	4.01E-07	5.31E-07	1.07E-06	1.33E-06	1.36E-06	**-5.69**	**-5.46**
Diuron		1.77E-06	3.95E-06	1.50E-06	3.55E-06	1.35E-06	3.27E-06	**-5.34**	**-4.97**
Ethofumesate	1.85E-05	7.44E-07	3.51E-05	4.32E-07	2.11E-05	7.07E-08	1.33E-05	**-5.90**	**-4.06**
Hexa(methoxymethyl)melamine									
Imidacloprid	2.19E-01	2.83E+00	2.11E+00	1.30E+00	1.24E+00	1.42E+00	3.34E+00	**0.74**	**0.84**
Mirtazapine									
Pendimethalin		4.17E-06		3.69E-06		4.24E-06		**-4.92**	
Phenylbenzimidazole sulfonic acid									
Propiconazole		1.10E-06						**-5.96**	
Propranolol				1.88E-04		5.61E-07		**-3.22**	
Roxithromycin				5.51E-07		6.57E-07		**-5.92**	
Tebuconazole		3.34E-05		2.27E-05		1.68E-05		**-4.14**	
Terbutryn	1.42E-07	2.51E-07	4.21E-07	3.28E-07	6.68E-07	3.58E-07	6.91E-07	**-6.03**	**-5.72**
Thiacloprid	2.47E-04	3.10E-04	4.85E-04	2.78E-04	3.44E-04	4.12E-04	2.83E-04	**-3.00**	**-2.87**
Tri(butoxyethyl) phosphate (TBEP)									
Triphenyl phosphate (TPP)	3.20E-05	5.16E-05	2.63E-05	1.81E-05	3.30E-06	1.57E-05	4.97E-06	**-4.07**	**-4.18**
Valsartan									
**log sumTU**	**-0.66**	**0.45**	**0.33**	**0.11**	**0.10**	**0.15**	**0.52**		

Table S11: Toxic Units for each compound and sumTUs for sampling sites. A value above -3.0 indicates the occurrence of chronic effects. All calculations are based on EC_{50} values from *D. magna*.

Substances	H1	W3		W4		W5		logTU	
	2017	2016	2017	2016	2017	2016	2017	2016	2017
1H-Benzotriazole		1.84E-04	4.05E-04	1.88E-04	5.16E-04	2.34E-04	7.06E-04	**-3.22**	**-2.79**
2-Benzothiazolsulfonic acid									
4+5-Methyl-1H-benzotriazole	6.10E-05	4.74E-05	2.51E-04	3.60E-05	2.82E-04	2.41E-05	2.98E-04	**-3.97**	**-3.05**
7-Amino-4-methylcoumarin									
7-Diethylamino-4-methylcoumarin									
Carbamazepine	8.66E-07	1.13E-06	2.64E-06	7.69E-07	6.14E-06	1.05E-06	7.33E-06	**-5.53**	**-4.77**
Carbendazim		1.44E-02						**-1.84**	
Cetirizin		2.78E-07		2.46E-07		3.11E-07		**-6.08**	
Citalopram	1.01E-07	8.76E-07	9.72E-07	8.62E-07	4.76E-07	1.11E-06	4.38E-06	**-5.54**	**-5.23**
Clarithromycin	6.34E-07	2.05E-07	4.01E-07	5.31E-07	1.07E-06	1.33E-06	1.36E-06	**-5.69**	**-5.46**
Diuron		1.77E-06	3.95E-06	1.50E-06	3.55E-06	1.35E-06	3.27E-06	**-5.34**	**-4.97**
Ethofumesate	1.85E-05	7.44E-07	3.51E-05	4.32E-07	2.11E-05	7.07E-08	1.33E-05	**-5.90**	**-4.06**
Hexa(methoxymethyl)melamine									
Imidacloprid	4.65E-05	6.01E-04	4.49E-04	2.76E-04	2.64E-04	3.02E-04	7.09E-04	**-2.93**	**-2.83**
Mirtazapine									
Pendimethalin		4.17E-06		3.69E-06		4.24E-06		**-4.92**	
Phenylbenzimidazole sulfonic acid									
Propiconazole		1.10E-06						**-5.96**	
Propranolol				1.88E-04		5.61E-07		**-3.22**	
Roxithromycin				5.51E-07		6.57E-07		**-5.92**	
Tebuconazole		3.34E-05		2.27E-05		1.68E-05		**-4.14**	
Terbutryn	1.42E-07	2.51E-07	4.21E-07	3.28E-07	6.68E-07	3.58E-07	6.91E-07	**-6.03**	**-5.72**
Thiacloprid	9.82E-07	1.23E-06	1.93E-06	1.11E-06	1.37E-06	1.64E-06	1.12E-06	**-5.40**	**-5.27**
Tri(butoxyethyl) phosphate (TBEP)									
Triphenyl phosphate (TPP)	3.20E-05	5.16E-05	2.63E-05	1.81E-05	3.30E-06	1.57E-05	4.97E-06	**-4.07**	**-4.18**
Valsartan									
**log sumTU**	**-3.79**	**-1.82**	**-2.93**	**0.11**	**-2.96**	**-3.22**	**-2.76**		

References

1. Mirco Bundschuh and Ralf Schulz, *Population response to ozone application in wastewater: An on-site microcosm study with gammarus fossarum (crustacea: Amphipoda).* Ecotoxicology, 2011. **20**(2): p. 466-473.
2. Caroline Naylor, Lorraine Maltby, and Peter Calow, *Scope for growth in gammarus pulex, a freshwater benthic detritivore.* Hydrobiologia, 1989. **188**(1): p. 517-523.
3. Pedro A Inostroza, Iván Vera-Escalona, Anna-Jorina Wicht, Martin Krauss, Werner Brack, and Helge Norf, *Anthropogenic stressors shape genetic structure: Insights from a model freshwater population along a land use gradient.* Environmental science & technology, 2016. **50**(20): p. 11346-11356.
4. CTA Moermond, *Environmental risk limits for pharmaceuticals: Derivation of wfd water quality standards for carbamazepine, metoprolol, metformin and amidotrizoic acid.* 2014.
5. Ana Rita R. Silva, Diogo N. Cardoso, Andreia Cruz, Joana Lourenço, Sónia Mendo, Amadeu M. V. M. Soares, and Susana Loureiro, *Ecotoxicity and genotoxicity of a binary combination of triclosan and carbendazim to daphnia magna.* Ecotoxicology and environmental safety, 2015. **115**: p. 279-290.
6. US FDA, *Retrospective review of ecotoxicity data submitted in environmental assessments for public display*. Docket.
7. Anne Munch Christensen, Sofie Faaborg-Andersen, Ingerslev Flemming, and Anders Baun, *Mixture and single-substance toxicity of selective serotonin reuptake inhibitors toward algae and crustaceans.* Environmental toxicology and chemistry, 2007. **26**(1): p. 85-91.
8. Marina Isidori, Margherita Lavorgna, Angela Nardelli, Luigia Pascarella, and Alfredo Parrella, *Toxic and genotoxic evaluation of six antibiotics on non-target organisms.* Science of The Total Environment, 2005. **346**(1-3): p. 87-98.
9. Susana Loureiro, Claus Svendsen, Abel LG Ferreira, Clara Pinheiro, Fabianne Ribeiro, Amadeu MVM %J Environmental Toxicology Soares, and Chemistry, *Toxicity of three binary mixtures to daphnia magna: Comparing chemical modes of action and deviations from conceptual models.* 2010. **29**(8): p. 1716-1726.
10. Laira L Damasceno de Oliveira, Sara Cristina Antunes, Fernando Gonçalves, Odete Rocha, and Bruno Nunes, *Acute and chronic ecotoxicological effects of four pharmaceuticals drugs on cladoceran daphnia magna.* Drug and chemical toxicology, 2016. **39**(1): p. 13-21.
11. Kyungho Choi, Younghee Kim, Jinyong Jung, Myung-Hyun Kim, Chang-Soo Kim, Nam-Hee Kim, and Jeongim Park, *Occurrences and ecological risks of roxithromycin, trimethoprim, and chloramphenicol in the han river, korea.* Environmental Toxicology and Chemistry: An International Journal, 2008. **27**(3): p. 711-719.
12. Kunde Lin, *Joint acute toxicity of tributyl phosphate and triphenyl phosphate to daphnia magna.* Environmental Chemistry Letters, 2009. **7**(4): p. 309-312.
13. Anne Bayer, Robert Asner, Walter Schüssler, Willi Kopf, Klaus Weiß, Manfred Sengl, and Marion Letzel, *Behavior of sartans (antihypertensive drugs) in wastewater treatment plants, their occurrence and risk for the aquatic environment.* Environmental Science and Pollution Research, 2014. **21**(18): p. 10830-10839.
